# Schistosome Vaccines for Domestic Animals

**DOI:** 10.3390/tropicalmed3020068

**Published:** 2018-06-19

**Authors:** Hong You, Pengfei Cai, Biniam Mathewos Tebeje, Yuesheng Li, Donald P. McManus

**Affiliations:** Molecular Parasitology Laboratory, QIMR Berghofer Medical Research Institute, Brisbane, QLD 4006, Australia; Hong.You@qimrberghofer.edu.au (H.Y.); Pengfei.Cai@qimrberghofer.edu.au (P.C.); Biniam.Tebeje@qimrberghofer.edu.au (B.M.T.); Yuesheng.Li@qimrberghofer.edu.au (Y.L.)

**Keywords:** schistosomiasis, *Schistosoma*, vaccine, zoonosis, Asia, Africa, domestic animals, buffalo, cattle, sheep, goats

## Abstract

Schistosomiasis is recognized as a tropical disease of considerable public health importance, but domestic livestock infections due to *Schistosoma japonicum*, *S. bovis*, *S. mattheei* and *S. curassoni* are often overlooked causes of significant animal morbidity and mortality in Asia and Africa. In addition, whereas schistosomiasis japonica is recognized as an important zoonosis in China and the Philippines, reports of viable schistosome hybrids between animal livestock species and *S. haematobium* point to an underappreciated zoonotic component of transmission in Africa as well. Anti-schistosome vaccines for animal use have long been advocated as part of the solution to schistosomiasis control, benefitting humans and animals and improving the local economy, features aligning with the One Health concept synergizing human and animal health. We review the history of animal vaccines for schistosomiasis from the early days of irradiated larvae and then consider the recombinant DNA technology revolution and its impact in developing schistosome vaccines that followed. We evaluate the major candidates tested in livestock, including the glutathione S-transferases, paramyosin and triose-phosphate isomerase, and summarize some of the future challenges that need to be overcome to design and deliver effective anti-schistosome vaccines that will complement current control options to achieve and sustain future elimination goals.

## 1. Introduction

As well as being a disease of great public health importance, schistosomiasis can also be a chronic debilitating infection of animals and a problem of considerable economic significance in Asia and many parts of Africa. It has been estimated, for example, that 165 million cattle are infected with schistosomiasis worldwide [1]. Schistosome infections of livestock are very important, if often underappreciated, and they are often causes of serious animal mortality and morbidity with several species incriminated. In South and South-East Asia, schistosomiasis is caused by the highly zoonotic *Schistosoma japonicum* and/or *S. mekongi*. *S. mattheei* and/or *S. curassoni* infect cattle, sheep and goats in southern and Central Africa, whereas *S. bovis* is a major veterinary problem in many Mediterranean and African countries, causing high levels of morbidity among susceptible ruminants (cattle, goats, sheep, horses, camels and pigs), resulting in considerably reduced economic output due to liver condemnation, reduced productivity, poor subsequent reproductive performance, increased susceptibility to other infectious agents, and death [1,2].

It has been shown also that uninfected animals grow and gain weight faster and are overall healthier than schistosome-infected animals. *S. bovis* has recently come into the spotlight as an emerging clinical health threat as well following the isolation of *S. haematobium*-*S. bovis* hybrids from children in Senegal [3] and after a recent schistosomiasis outbreak in France [4]. There are also reports of eggs indicative of potential *S. haematobium*-*S. mattheei* hybrids in Zimbabwe and in southern African ruminants [5]. The hybridization between human and ruminant schistosomes is of particular importance as inter-species hybridization may have a considerable impact on parasite evolution, disease dynamics, transmission rates and control interventions. Laboratory hybrids acquire enhanced characteristics, including infectivity, growth rates, maturation and egg production and, in cattle, it has been reported that introgressive *Schistosoma* hybrids may affect the success of drug treatment and can cause severe disease outbreaks [6]. The zoonotic component of transmission in sub-Saharan Africa does appear to be more significant than previously assumed, and may thereby affect the recently revised WHO vision to eliminate schistosomiasis as a public health problem by 2025. Moreover, animal schistosomiasis is likely to be a significant cost to affected communities due to its direct and indirect impacts on livelihoods. These findings underscore the need for improved disease control in animals, to reduce the zoonotic transmission of *S. japonicum* and *S. mekongi*, and to prevent the spread of hybrid schistosomes to humans from animal reservoirs.

The deployment of suitable anti-schistosome vaccines for use in animals has long been advocated as part of the solution to control and eliminate schistosomiasis. Indeed, a vaccine-directed control program that reduces prevalence, intensity and transmission of the disease in animals can directly benefit both humans and animals in endemic sites and improve the local economy, features that are in clear alignment with the One Health concept synergizing human and animal health. Furthermore, genuine change of the disease spectrum in endemic areas demands lasting results and the development and positioning of an effective vaccine represents an option for long-term protection. An entirely vaccine-based approach targeting humans and animals for schistosomiasis control is unrealistic, but acceptable protection could be achieved by chemotherapy treatment followed by vaccination aimed at reducing, or markedly delaying, the development of pathology and morbidity and limiting the impact of re-infection [7,8]. Thus, the issue is not drugs competing with vaccines, but how to graft a vaccine approach onto current schistosomiasis control programs [7,8]. Mathematical modelling of schistosome transmission supports this concept, predicting that a schistosome vaccine capable of reducing the faecal egg output in bovines by 45% in conjunction with praziquantel treatment would lead to a significant reduction in the transmission of schistosomiasis japonica almost to the point of elimination [9,10]. Domesticated animals represent significant reservoirs of *S. japonicum*, and their vaccination offers an approach to control schistosomiasis by interrupting its zoonotic transmission. Pertinent to this are studies undertaken in China [11] and the Philippines [12], which showed that bovines, in particular, are major animal reservoir hosts for *S. japonicum*, responsible for up to 90% of environmental egg contamination.

## 2. The Early Days—Irradiated Larval Vaccines

The immunological control of animal schistosomiasis was first advanced as an option in the 1970s as in many endemic areas, particularly in Africa, the use of molluscicides or chemotherapy as interventions was either too expensive or impractical [13]. Further, cattle had been shown to develop partial immunity to reinfection with *S. bovis* and *S. mattheei*, and sheep had been shown to develop some resistance to reinfection with *S. mattheei* [13]. As well, results of experiments in calves, cattle and sheep with harmless heterologous schistosome species suggested that the presence of adult egg-producing worms was not necessary for the development of acquired immunity to reinfection; these observations suggested the possibility of developing a vaccine incorporating either non-pathogenic heterologous larvae or, alternatively, larvae of the homologous species attenuated by irradiation to prevent them reaching full maturity [13]. Cercariae and schistosomula, attenuated with gamma rays, X-rays, or ultraviolet (UV) irradiation, were subsequently reported to elicit protective immunity against schistosomiasis. Indeed, the radiation attenuated vaccine approach provided protective efficacy against *Schistosoma* species in many different host species, including mice, rats, chimpanzees and baboons. The radiation attenuated vaccine also proved to be highly effective in livestock (cattle, buffaloes, sheep and pigs), thereby establishing the potential of developing irradiated live schistosome veterinary vaccines in the laboratory and their extended application to the field.

In a pioneering study in 1976, Taylor et al. [13] demonstrated that sheep could be partially protected against *S. mattheei* by prior immunisation with live cercariae or schistosomula of *S. mattheei* irradiated at 6 krad by a [^60^Co] source; and the study showed that effective immunisation was not dependent on the presence of a mature worm infection or on cercarial penetration of the skin by the immunising infection, as artificially transformed schistosomula were as immunogenic when injected into sheep as cercariae that penetrated the skin. The results opened up the possibility of making a live vaccine against ovine schistosomiasis, with the caveats that the problem of live parasite storage needed to be overcome and that a more efficient immunising schedule was needed. Other reports described highly effective immunisation of sheep with irradiated *S. bovis* cercariae [14] and irradiated schistosomula [15].

The first attempts at immunising cattle (zebu calves) were against *S. bovis* in Sudan and involved vaccination with 3 krad-irradiated homologous larval parasites first under experimental laboratory challenge using irradiated schistosomula or cercariae [16], and then in the field, under natural challenge when the calves (50% immunised with irradiated schistosomula) were allowed to graze in a *S. bovis-*endemic area for 10 months [17]. In both trials, vaccination was safe and, compared with control animals, the vaccinated animals had higher growth rates and significantly fewer adult worms and tissue and faecal eggs, indicating that zebu cattle could be protected effectively against *S. bovis* by vaccination with irradiated parasites.

An inherent problem in the production of a potential live radiation-attenuated vaccine at this time was the limited shelf-life of the attenuated parasites but new techniques to successfully cryopreserve schistosomula, including vitrification (solidifying a liquid without crystal formation) [18], were developed by Eric James and his team, which enabled their indefinite storage. In 1985, a cryopreserved homologous radiation-attenuated schistosomula vaccine evoked a good level of protection in sheep against *S. bovis* challenge [19].

At about the same time, effective irradiated vaccines were also being developed in China against *S. japonicum* infections, first in mice, and then in domestic pigs, sheep, cattle and buffaloes. These livestock animals, particularly water buffalo (*Bubalus bubalis*), represent important reservoirs of *S. japonicum*, so their vaccination provides a highly suitable approach to control schistosomiasis by zoonotic transmission interruption. The initial experiments were undertaken by Hsü and his team and were reported in 1983 [20] and 1984 [21]. The first study [20] used experimental infections with cattle calves receiving a vaccine of approximately 10,000 irradiated schistosomula followed by a challenge with 500 normal cercariae; the best results, in terms of reduced worm counts, involved three injections (partly given intramuscularly and partly intradermal) of larvae irradiated with 36 krad. The second study [21] was essentially a repeat with calves being perfused later (54–57 days as opposed to one month in the earlier experiment), thereby allowing the infection to mature to egg production, although the reduction in worm numbers was similar. The efficacy of this irradiated vaccine was field tested in vaccinated yearling cattle that were naturally challenged in an area in China highly endemic for schistosomiasis [21]; at perfusion, significant reductions in worm burden and liver eggs were recorded in the vaccinees compared with controls.

Although the work was undertaken in 1979 and 1980, Xu et al. [22] reported in 1993 on trials in bovines of vaccinations with single or double doses of 10,000 or 20,000 cryopreserved irradiated (CI) (20 krad) *S. japonicum* schistosomula together with 1 mL bacille Calmett-Guérin (BCG); worm numbers were reduced by 55–65% in vaccinated water buffalo (*Bubalus bubalis*) and cattle calves after being challenged with 500–1000 cercariae. A field test of the vaccine (10,000 CI schistosomula) resulted in a worm reduction of 53% in vaccinated water buffalo (*Bubalus bubalis*) calves [22]. Intradermal vaccination with 30,000 freeze-thaw (FT) schistosomula and BCG by the same group provided 57% protection in cattle. Similar levels of protection were obtained in sheep immunised with CI and FT schistosomula following challenge with 500 cercariae [23].

Cercariae have also been used as the irradiated vaccine source. Shi et al., in a report published in 1990 [24], vaccinated water buffaloes three times with 10,000 *S. japonicum* cercariae irradiated with a cheap, simple and portable UV light source at a dose of 400 μW min/cm^2^. A challenge infection of 1000 untreated cercariae was given to vaccinated and naive control animals; the experiment was terminated six weeks post-challenge and, compared with controls, the vaccinated animals developed 89% resistance to infection with *S. japonicum.* Using a similar vaccination regimen, the same group undertook a study in pigs with a challenge infection of 1000 untreated cercariae given 2.5 or 6 months after the last immunization, with age-matched naïve pigs challenged as controls; immunized animals developed 90% resistance against the challenge [25].

The use of live schistosomula or cercariae vaccines attenuated by ionizing radiation or by biochemical means [26] potentially provides a method to protect domestic livestock against infection with schistosomes, but they have generally not found favour as a practical means of vaccinating domestic animals in the field (or humans) and they have never been used on a large scale. The reasons for this include: (1) the high production costs and labour-intensive efforts necessary to obtain the large numbers of cercariae required from infected snails; (2) the difficulties in standardizing the dose of ionizing radiation in order to induce larval attenuation and to ensure irradiated parasites do not retain some level of infectivity, thereby causing a breakthrough infection if insufficiently irradiated; (3) the requirement for cryo-preservation in order to store and then transport attenuated parasites over long distances; and (4) the safety issues associated with potential toxicity of administering a live vaccine and local inflammatory responses at the site of vaccination.

Thus, although in general the initial successes with irradiated schistosome vaccines were encouraging, with the research benefitting from substantial funding support during the 1970–1990s, progress stalled thereafter. This was mainly due to a change in research emphasis when the new advances in recombinant DNA technology were applied for the development of schistosome vaccines. Consequently, limited work has been undertaken on livestock animals with live attenuated schistosome vaccines in the past 25 years. In one approach protocols were established to compare the level of protection induced by recombinant and naked plasmid DNA formulations with the gold standard gamma or UV-irradiated cercarial vaccines in pigs [27,28]. Another avenue involved systematically investigating cellular and humoral immune responses generated by the protective UV-attenuated *S. japonicum* cercarial vaccine in pigs in order to identify key molecules involved in the process leading to resistance, thereby providing a paradigm for the development of an optimal vaccine formulation for both veterinary and clinical application [25,29,30,31,32,33].

## 3. The Impact of Recombinant DNA Technology on the Development of Animal Vaccines for Schistosomiasis

As indicated, in efforts to circumvent the perceived problems of using live, attenuated *S. japonicum* schistosomula or cercariae as vaccines to prevent infection in domestic livestock, and with the new techniques in genetic manipulation fast making ground, the research focus changed. Many groups attempted to reproduce or even improve on the protection afforded by substituting native antigens or chemically-defined schistosomal antigens genetically engineered in bacteria or yeast as recombinant proteins, or as plasmid DNA vaccines. The three most tested molecules tested in livestock have been glutathione S-transferases, paramyosin and triose-phosphate isomerase, details for which now follow.

### 3.1. The Glutathione S-Transferases

Two of the first schistosome molecules to be cloned and expressed (first in *E. coli* and then yeast) were the 28 kDa glutathione-S-transferase from *S. mansoni* (28 GST, P28 or Sm28) [34] and the 26 kDa GST homologue (termed Sj26) from *S. japonicum* [35]. The molecular cloning of the genes encoding the 28 GSTs of *S. bovis*, *S. japonicum* and *S. haematobium* soon followed [36]. The GSTs are enzyme isoforms that catalyse the detoxification of lipophilic molecules by thiol-conjugation. They were considered attractive vaccine candidates in ruminants and other mammals because it was hypothesised that antibody-mediated neutralization of this detoxification function could render the schistosome vulnerable to toxic products generated by immune attack at the tegument or in the gut [37]. Accordingly, it was shown that native GST and GP38, which shares protective epitopes with keyhole limpet haemocyanin (KLH), exhibited vaccine potential against *S. mansoni* in small animal experiments [34]. Subsequently, native forms of *S. bovis* GST and KLH were tested for vaccine efficacy against *S. bovis* in Zebu cattle, and this resulted in specific antibodies being generated against both molecules and, depending on the vaccination schedule, significant reductions in faecal and tissue eggs also resulted; notably, however, vaccination did not reduce adult worm numbers [38]. In contrast, whereas vaccination of goats [39] and sheep [40] with recombinant *S. bovis*-derived 28GST (recombinant *S. bovis* 28GST), followed by experimental challenge with *S. bovis* cercariae, resulted in reduced worm burden, there was no impairment of fecundity. In a further report, immunization of calves with recombinant *S. bovis* 28GST induced significant reductions in female worm numbers, faecal eggs counts, and the number of viable eggs (determined by miracidial counts), in animals exposed to natural *S. mattheei* infection in the field [41]. In contrast, the same immunization schedule generated no protective effect against a massive (10,000 cercariae) single experimental challenge with *S. mattheei.* Being highly susceptible to *S. mattheei* infection, calves produce high parasite recovery rates, and the effect of vaccination and the generated immune response may have been insufficient to protect the animals, raising questions of the biological relevance of massive experimental challenges in the evaluation of protective immunity to schistosomiasis [41,42].

Subsequently, although considerable work on the schistosome GST vaccines focused on *S. mansoni* and then *S. haematobium*, it was clear that the very significant inhibition of female worm fecundity and egg viability was the most evident host protective effect generated against the GSTs of all schistosome species, including *S. japonicum*, by homologous vaccination [43]. The exact mechanism whereby anti-GST antibodies affected egg production and viability remained unanswered, but the phenomenon appeared to be associated with their inhibition of the enzymatic activity of GST, the consequent impairment in prostaglandin biosynthesis (prostaglandin D2 orchestrates various stages of the host immune response), and the resultant effect on schistosome biology, including a reduction in female worm fecundity [44].

Following on from the work with *S. mansoni* GSTs, partial protection was recorded in Chinese sheep and bovines vaccinated with *S. japonicum* 28 kDa GST (Sj28GST), either as a recombinant protein or plasmid DNA vaccine [45,46,47], but most reports were of the vaccine potential of the 26 kDa GST isoform (Sj26GST) in different mammalian hosts [48,49] (Table 1). Recombinant (r) Sj26GST induced a prominent anti-fecundity effect, as well as a significant, albeit moderate, level of protection in terms of reduced worm burdens in sheep, cattle and pigs, following challenge infection with *S. japonicum* [48,49,50,51,52]. Similar levels of vaccine efficacy were obtained in water buffaloes vaccinated with purified rSj26GST [50,52]. Anti-Sj26GST antibodies were generated in the immunized water buffaloes and, following challenge with *S. japonicum*, the typical anti-fecundity effect was observed with decreased numbers (circa 50%) of faecal eggs and eggs deposited in host livers and intestines [52]. As well, the rSjc26GST vaccine reduced the egg-hatching capacity of *S. japonicum* eggs into viable miracidia by nearly 40% [52]. Encouragingly, field trials demonstrated that the protective effect of rSj26GST against *S. japonicum* could be maintained in cattle and water buffaloes for at least 12 months post-vaccination [53,54], but whereas research to improve efficacy has continued using the murine model of schistosomiasis japonica (e.g., [55]), it is disappointing that there have been no further trials of the vaccine in livestock animals. The picture is somewhat brighter in regards to paramyosin, with some of the early work and recent progress on this vaccine candidate now being described.

### 3.2. Paramyosin

Paramyosin is a large (97 kDa) coiled-coil myofibrillar protein restricted to invertebrates. In schistosomes it is found on the tegumental surface of lung stage schistosomula, in the cercarial secretory glands, and in the muscles of adult worms and larvae [60]. This molecule first generated interest as a vaccine candidate based on results of experiments in mice targeting *S. mansoni* [60]. Trials in sheep with native and an expressed and purified recombinant fragment of Chinese strain *S. japonicum* paramyosin (Sj-97) resulted in significant partial protection being obtained [45]. Subsequently, vaccine experiments in pigs [27] and water buffaloes [61], using full length Chinese *S. japonicum* paramyosin, yielded further encouraging protective efficacy against challenge infection, although the protection was again only partial. At this time (the early 2000s) the limitations to wide-spread use of recombinant (r) Sj-97 as a vaccine were the size of the protein, the inadequate expression levels obtained, resulting in low yields of the recombinant protein, its insolubility in yeast and bacterial expression systems, the inevitable costs of its up-scaled production and purification, and the funds required for large-scale buffalo vaccine trials in the field; this resulted in a halting in its progression as a priority vaccine candidate.

In 2008, however, Jiz et al. [62] published a new robust method for the pilot-scale expression and purification of rSj-97 by its extraction from *E. coli* inclusion bodies and purification with sequential anion-exchange, hydroxyapatite, and size exclusion chromatography. The purified protein was greater than 95% pure, free of significant endotoxin contamination, adopted an alpha-helical coiled-coil tertiary structure, bound immunoglobulin and collagen, and represented a significant step forward towards preclinical evaluation of paramyosin as a vaccine for schistosomiasis [63]. The same team showed that the rSj-97 vaccine, adjuvanted with Montanide ISA 206, was safe, well tolerated and robustly immunogenic among water buffaloes resident in four villages in Leyte, the Philippines, an area highly endemic for schistosomiasis japonica [64]. They conducted three vaccine trials (in 2008, in 2013 and in 2016) to assess the level of protection generated in water buffaloes with the rSj-97 vaccine; animals received and tolerated well three doses (250 μg/dose or 500 μg/dose) of rSj97 plus Montanide ISA 206 at 4 weekly intervals before a challenge infection with 1000 *S. japonicum* cercariae [58]. The 3 × 250 μg/dose of rSj97 (2008 and 2013 trials) did not result in a significant reduction in worm burden in vaccinated animals compared with controls injected with ISA 206 emulsified with lyophilisation buffer, but the increased dosage (3 × 500 μg/dose of rSj97) in the 2013 and 2016 trials resulted in a significantly lower worm burden (by 57.8% in both trials) in vaccinated water buffaloes compared with controls [58]. The trialled rSj97/ISA206 vaccine resulted in a mixed immune response with induction of both IgG1 and IgG2 anti-paramyosin antibodies, but no conclusive correlation was found between isotype distribution and protection [58]. The group also reported undertaking a trial in buffaloes with the rSj97-ISA206 vaccine followed by six months of community-based field exposure after vaccination [58], but the results have not yet been released.

### 3.3. Triose-Phosphate Isomerase (TPI)

Another leading anti-schistosome vaccine candidate is triose-phosphate isomerase (TPI). Following its uptake in schistosomes, glucose is metabolised via glycolysis to provide critical energy in the form of ATP to ensure parasite survival; TPI is a highly efficient enzyme and pivotal in this process, converting glyceraldehyde-3-phosphate to dihydroxyacetone phosphate. It is located in most cells of the adult schistosome and on the surface membranes of newly transformed schistosomula, the stage in the mammalian host that is considered the most likely target of an anti-schistosome vaccine [50]; a plasmid DNA vaccine encoding *S. japonicum* Chinese strain TPI (SjCTPI) was shown to reduce worm burdens in mice and pigs [57,65]. Subsequently, the efficacy of SjCTPI and the 23 kDa integral membrane protein tetraspanin (SjC23), independently, and as fusions with bovine heat-shock protein 70 (Hsp70), co-administered with an interleukin-12-expressing plasmid as adjuvant, were assessed as DNA vaccines in China in water buffaloes against experimental *S. japonicum* challenge [10]. The most encouraging vaccine was the SjCTPI-Hsp70 construct, which reduced worm burdens by 51.2%, reduced liver eggs by 61.5%, reduced faecal eggs by 52.1% and resulted in a 52.1% reduction in hatching of faecal miracidia [10]. The SjCTPI-Hsp70 vaccine has subsequently undergone field testing in bovines against natural *S. japonicum* challenge in China. The trial comprised a four-year double-blind cluster randomised intervention in 12 administrative villages around the schistosomiasis-endemic Dongting Lake in Hunan Province, designed to assess the impact of a multi-component integrated control strategy, including bovine vaccination using a heterologous ‘prime-boost’ delivery of the SjCTPI vaccine, on schistosome transmission. The study design and baseline results have been published [66]. In brief, the integrated control strategies included: (a) Combined human mass chemotherapy and the bovine SjCTPI vaccine or placebo vaccine; (b) Combined mollusciciding of snails and bovine SjCTPI vaccine or placebo vaccine; (c) No other intervention (treatment of infected individuals only) and bovine SjCTPI vaccine or placebo vaccine. The primary end-point for the study is human infection rate, with secondary endpoints including bovine infection rate, snail infection prevalence and the density of infected snails.

## 4. Challenges and Opportunities for the Future

In summary, many of the *S. japonicum* vaccines tested in domestic livestock include enzymes, muscle components and membrane proteins (Table 1) [67,68]. DNA vaccines have found particular appeal as they generate both T-cell and B-cell (or antibody-mediated) immune responses, their preparation and production are convenient and cost-effective, and they can even be used in the field without a cold chain. Another advantage of applying DNA vaccines compared with other approaches is the possibility of targeting the in vivo expressed recombinant antigen to different cell compartments. Furthermore, methods such as prime-boost regimens and the use of adjuvants (such as IL-12) in combination with a DNA vaccine can enhance its protective efficacy.

Other than some proteomic analyses of *S. bovis* and the *S. bovis*-host interface, aimed at identifying potential new vaccine and drug targets [69], it is surprising that no recent research has been reported in the development and testing of different vaccines targeting schistosomiasis infections caused by *S. bovis* and *S. mattheei* in livestock, despite the early encouraging successes. This may be somewhat short-sighted as it has been recently argued that animal schistosomiasis likely causes a considerable drain, both directly and indirectly, on the local economy of many poor communities in sub-Saharan Africa [70]. Further, the zoonotic component of schistosomiasis transmission in Africa appears to be far more significant and important than previously thought [6], and this may impact on the current WHO vision to eliminate schistosomiasis as a public health issue by 2025. Consequently, it has been considered that extending treatment to include animal hosts in a One Health approach, as has been successfully undertaken in China, would be both of economic and public health benefit. The positioning of an animal-based transmission blocking vaccine as part of an integrated control package fits well into this scenario, as was recognised as an outcome of two workshops co-sponsored by the Bill and Melinda Gates Foundation and the National Institute of Allergy and Infectious Diseases on schistosomiasis elimination strategies and the potential role of a vaccine [71,72]. Some proposed Preferred Product Characteristics for a clinical versus a veterinary vaccine against schistosomiasis were formulated at one of these workshops, indicating that the safety profile of the latter is more straightforward and less rigorous [71]. Whereas this characteristic could potentially reduce the costs of deploying a schistosomiasis vaccine for use in livestock as opposed to one for human application, an evaluation of the economics and benefits of applying the two different types of vaccine needs to be undertaken.

One significant challenge impacting on the future development of veterinary-targeted vaccines to aid in the control of schistosomiasis continues to be our rather scanty knowledge of the immunology of schistosome infections in natural livestock hosts, especially bovines, compared with experimental model animals, such as mice. This is due, in part, to the high cost of purchasing and maintaining large animals for immunobiological studies and the scarcity and high price of immunological reagents for studying the relevant immune responses. Nevertheless, some recent research has provided new insights on immunity against schistosomiasis japonica in field-exposed natural hosts such as cattle and water buffalo, particularly the immune response generated against migrating schistosome larvae, the likely targets of an anti-schistosome vaccine. In an important study, McWilliam et al. [73] investigated the immune response evoked in the lungs and skin, the major sites of larval migration, in previously *S. japonicum*-exposed and re-challenged water buffaloes. A powerful allergic-type response was generated in the skin with IL-5 transcript levels being elevated, with IL-10 levels decreased. In addition, a Th1 type immune profile was demonstrated in stimulated cells from the lung-draining lymph node, whereas a predominant Th2 type immune response was apparent in stimulated cells from the skin-draining lymph node; these immune responses reflected the timing of parasite migration and occurred consecutively. The intense Th2 type profile generated at the cercarial penetration site differs markedly from that evident in mice, suggesting a possible mechanism of immunity. Furthermore, the study suggested that a reduced/delayed immune response occurred in buffaloes challenged with high numbers of cercariae compared with lower numbers, particularly in the skin.

In tandem with studies comparing immune responses in buffalo (in which age-related resistance likely occurs) and yellow cattle (in which it does not) against schistosomiasis [74,75], and further immunological exploration of the self-cure effect in older buffalo and other livestock hosts such as pigs against schistosomes [76], the investigation by McWilliam et al. [73] provides a unique, in-depth appreciation of the immunobiology of schistosomiasis in a natural host. Such studies can aid in the design and delivery of more effective anti-schistosome vaccines that will complement current control options so as to achieve and sustain future elimination goals.

## Figures and Tables

**Table 1:** *S. japonicum* vaccines tested in domestic livestock.

Host	Antigen	Method of Immunization Regimen	Vaccine Efficacy			Ref
			Worm Burden Reduction %	Liver Egg Burden Reduction %	Fecal Egg Reduction %	
Pig	UV-attenuated cercariae	Single/three immunizations	59–78	89	86–99.7	[31]
	Irradiated cercariae		>95	>95	>95	[28]
	SjC23-pcDNA3.1 DNA	Conjunction with/without IL-12	29–58	48–56		[56]
	SjTPI-pcDNA3.1 DNA	Conjunction with/without IL-12	46–48	49–66		[57]
	Paramyosin	Conjugated with alum or TiterMax	32–34			[27]
Water buffalo	Paramyosin	Montanide ISA 206	52–58			[58]
	SjCTPI DNA	Fused to bovine heat shock protein 70	41–51	42–62	33–52 (miracidial hatching)	[10]
	SjC23DNA	(Hsp70) and boosted with a plasmid DNA encoding IL-12	45–51	43–54	47–52 (miracidial hatching)	
	Sjc26GST (field study)	Twenty months after vaccination, the infection rate in buffaloes was reduced by 60–68%				[53]
	Sjc26GST		22	50	50	[52]
	Sj28GST (field study)	Injected with Freund’s adjuvants	37	33	62 (miracidial hatching)	[46]
	Cryopreserved-irradiated and freeze-thaw schistosomula	Single/twice intradermal vaccination	62–65			[22]
	Cercariae UV irradiated	Six immunizations	89			[24]
Goat	Sj31 and Sj32	DNA priming-protein boosting	21–32	47–52	48–54 (miracidial hatching)	[59]
Cattle	Sj26GST		30	60		[54]
	Sj28GST-DNA	Three immunizations	44	19	77	[47]
	Sj23-DNA		33	34	66	
	Sj28GST + Sj23-DNA		38	48	68	
Sheep	Sj26GST	Injected with Freund’s adjuvants	54–62	42–55	11–38	[45,46]
	Sj28GST		61–69	59–69	43–60	
	Sj23		58–66	56–66	40–58	
	Native Paramyosin			68	43	
	Paramyosin			56	16	
